# Stable association of a chlamydial symbiont with the freshwater predator *hydra* suggests broad host potential

**DOI:** 10.1093/ismejo/wrag104

**Published:** 2026-04-30

**Authors:** Angelika Schwarzhans, Justine Boutry, Jácint Tökölyi, Norbert Cyran, Martin Kunert, Matthias Horn, Astrid Collingro

**Affiliations:** Centre for Microbiology and Environmental Systems Science, University of Vienna, Djerassiplatz 1, Vienna 1030, Vienna, Austria; Doctoral School in Microbiology and Environmental Science, University of Vienna, Djerassiplatz 1, Vienna 1030, Vienna, Austria; CREEC/CANECEV (CREES), MIVEGEC, Unité Mixte de Recherches, IRD 224–CNRS 5290–Université de Montpellier, 911 avenue Agropolis, 34394 Montpellier Cedex 05, Hérault, France; MTA-DE “Momentum” Ecology, Evolution & Developmental Biology Research Group, Dept. of Evolutionary Zoology, University of Debrecen, Egyetem tér 1, Debrecen 4032, Hajdú-Bihar County, Hungary; Faculty of Life Sciences, Core Facility for Cell Imaging and Ultrastructure Research, University of Vienna, Djerassiplatz 1, 1030 Vienna, Vienna, Austria; Centre for Microbiology and Environmental Systems Science, University of Vienna, Djerassiplatz 1, Vienna 1030, Vienna, Austria; Centre for Microbiology and Environmental Systems Science, University of Vienna, Djerassiplatz 1, Vienna 1030, Vienna, Austria; Centre for Microbiology and Environmental Systems Science, University of Vienna, Djerassiplatz 1, Vienna 1030, Vienna, Austria

**Keywords:** Chlamydiae, Hydra, symbiosis, endoderm, vertical transmission

## Abstract

Symbiotic associations between microorganisms often involve eukaryotes partnering with microbes for nutrient exchange, protection, and resource acquisition. Bacterial lineages like the *Chlamydiota* have evolved entirely symbiotic lifestyles, exploiting their eukaryotic hosts for energy, diverse metabolites, and shelter. The study of environmental chlamydiae—outside the well-studied vertebrate host range—has revealed diverging interactions on the mutualism–parasitism spectrum. This highlights their potentially important roles in host–microbe interactions underscoring the relevance of obtaining isolates from diverse environments and hosts. Here, we describe an isolate of a chlamydial symbiont of the freshwater cnidarian *Hydra.* The symbiont could be isolated and stably maintained in insect cell lines and represents a member of the recently described family-level lineage Chlamydiae Clade III for which we propose the name *Endochlamydiaceae*. Fluorescence and electron microscopy reveal the symbiont morphology and its endodermal location. Comparative genomics shows the isolate, named *Endochlamydia hydrae*, encodes a conserved set of genes involved in host invasion, communication, and pathogenicity. Instead of displaying unique genomic adaptations to its animal host, *E. hydrae* shows signs consistent with ongoing genome reorganisation and streamlining, suggesting a more recent host shift. Screening for closely related 16S rRNA gene sequences in public environmental microbiomes also indicates a broader host range. Moreover, exploration of environmental *Hydra oligactis* populations revealed they might serve as host for a wider spectrum of chlamydial species. This study highlights the evolutionary success of chlamydiae and their genomic toolkit to infect a wide range of hosts and their ecological significance by interacting with diverse organisms.

## Introduction

Symbiotic associations shape ecosystems as well as lifestyles and evolution of organisms thriving within them. Many of these associations are ancient and provide good opportunities to study the dynamics of processes influencing them. Members of the bacterial phylum *Chlamydiota* that have been living an obligate endosymbiotic lifestyle for the past 1–2 billion years [1, 2] are found associated with hosts from many eukaryotic clades, especially as important human and livestock pathogens, and also have been detected in various environments [3]. They thrive as symbionts of a diverse range of protist hosts, but also as symbionts of arthropods in soil and in cnidarian hosts in marine habitats [3–7].

Chlamydiae alternate between two metabolically different stages, a replicative stage (reticulate body, RB), typically residing inside host derived vacuoles termed inclusions, and a non-replicating, infectious stage (elementary body, EB), permitting extracellular survival and reinfection [8–10]. The chlamydial dependence on eukaryotic host organisms and the difficulty of isolating and maintaining many hosts in a laboratory, hampers the cultivation of novel chlamydial isolates [11, 12]. Thus, genomics and metagenomics are often the methods of choice when studying environmental chlamydiae. Although chlamydiae generally have small genomes of around 1–3 Mbp and lack many genes necessary for essential metabolic pathways, a comparatively large core gene set is conserved throughout all chlamydial clades [1]. This toolkit helps them to not only colonize and manipulate host organisms, but also to exploit them for energy, amino acids, and nucleotides. This includes genes like the highly conserved type III secretion system (T3SS) for host cell manipulation and nucleotide transport systems, which allow them to complement for their lack of de novo nucleotide synthesis and simultaneously facilitate energy parasitism [7, 13, 14].

The cnidarian freshwater predator *Hydra* has been well documented for its close association with other organisms. A thoroughly investigated example are *Chlorella* algae in *Hydra viridissima,* showing a close association and co-evolution between the two partners [15]. Symbiotic associations in *Hydra* have also been shown to be essential in the development and feeding behaviour of *Hydra* polyps [16, 17]. Another example involves a *Hydra oligactis* strain for which spontaneous formation and persistence of tumors was only observed in the presence of a bacterial conflict between *Pseudomonas* and the spirochete *Turneriella* [18]. More recent findings on the microbiome of several different *Hydra* species have contributed to the understanding of innate immunity through antimicrobial peptides by controlling resident bacteria [19], which in turn provide resistance against colonisation [20]. Medusozoa like *Hydra* are amongst the first animal lineages to possess Toll-like receptor-like (TLR-like) proteins, nucleotide-binding oligomerization domain-like receptors (NLR)-like proteins, and a basic NF-kB pathway [21]. Thus, *Hydra* is used as a model organism to study the evolution of the innate immune system. *H. oligactis* symbiosis with bacteria of the phylum *Chlamydiota* has been suggested in a previous study, which investigated wild *Hydra* populations from Hungary and France [22].

In this study we present a closer description and analysis of this *Hydra*–chlamydiae symbiosis, derived from wild populations. By combining fluorescence- and transmission electron microscopy with genome sequencing and analysis, we characterize an isolate of a member of the recently proposed Chlamydiae Clade III (CC-III) family [1]. Both genomic analysis and environmental distribution of this chlamydial species point to an evolutionarily recent switch to *Hydra* as a host, thus offering an exciting study model for host-symbiont evolution and the adaptation to host immune systems of two ancient organism lineages.

## Materials and methods

### Maintenance of *Hydra* populations in the lab

*Hydra* species and strains were provided by Frédéric Thomas and had been derived from cultures collected in April 2021 [22]. These included *H. oligactis* strains St. Petersburg (SpB), X11/14, C2/7, Montaud and *Hydra vulgaris* [22]. The animals were maintained at 18°C in separate 200 ml glass beakers for each strain, containing sterilized Volvic water, with a photoperiod of 12 h. They were fed 2–3 times per week with *Artemia salina* nauplii. Nauplii were obtained as previously described [23], using 400 ml of seawater (Classic Sea Salt, Tropic Marin, at 32–33 ppm). Each feeding of *Hydra* cultures was followed by a water change 4–8 h post-feeding.

### DNA isolation, PCR, and symbiont quantification

To check for chlamydial presence, 68 polyps were tested from *H. oligactis* strains SpB, X11/14, C2/7, Montaud, and *Hydra vulgaris*. For DNA extraction from individuals or culture supernatant the DNeasy Blood and Tissue Kit for DNA Isolation (Qiagen) or the InnuPREP DNA mini kit (IST Innuprep) were used according to the manufacturer protocols (Supplementary Table S1). For DNA isolation from culture supernatant 30 ml of supernatant was collected from seven *H. oligactis* Montaud cultures (Supplementary Table S2).

The presence of chlamydiae in individual animals was first determined with PCR using the chlamydiae-specific primer pair SigF2 (=Chl40F) (5’ CRGCGTGGATGAGGCAT 3′; [24]) and Chl523R (5’ CCYYMCGTATTACCGCAGCT 3′) targeting the 16S rRNA gene in a T100 Thermo Cycler (BioRad) under the following cycling conditions: Initial denaturation at 95°C for 3 min, 35 cycles with 95°C for 30 seconds, 64.5°C for 30 seconds, 72°C for 1 min, and final elongation at 72°C for 5 min, using the DreamTaq Green PCR Master Mix (Thermo Scientific). 25 individuals were sequenced with Sanger sequencing at Microsynth AG (Austria). To quantify chlamydiae digital PCR (dPCR) was performed using the QIAcuity EvaGreen PCR kit (Qiagen) and the chlamydiae-specific primer pair described above on a QIAcuity One digital PCR device (Qiagen) as recommended by the manufacturer. The cycling conditions were 95°C for 2 min; 40 cycles of 95°C for 30 seconds, 64.5°C for 45 seconds, 72°C for 1 min; 40°C for 5 min with imaging at an exposure time of 350 ms and a gain of 3. Direct quantification data were acquired with the QIAcuity Software Suite 2.5.0.1 (Qiagen). Chlamydial 16S rRNA gene copy numbers per *Hydra* or ml supernatant, respectively, were calculated based on these data and divided by two to obtain genome copy numbers. Cell numbers above 100 chlamydiae per *Hydra* were considered to be positive.

### Histological sample processing

Four individuals of symbiotic *H. oligactis* Montaud polyps were incubated in a 3% (w/v) formaldehyde solution in Volvic water for 4 h at 4°C. The liquid was carefully removed and animals were washed 3x with PBS (40 g l^−1^ NaCl (Roth), 9 g l^−1^ Na_2_HPO_4_*2H_2_O (Roth), 1.2 g l^−1^ KH_2_PO_4_ (Sigma), 1 g l^−1^ KCl (Millipore)). Fixed animals were stored in PBS for no longer than a day at 4°C. Animals were embedded in paraffin and cut into 4 μm thin sections at the Vienna Biocenter Core Facility Histology. Dewaxing of the sections on the slide was performed directly before processing the slides for fluorescence *in situ* hybridisation (FISH) (described below). Slides were incubated three times for 10 minutes in Roti Histol (Roth), followed by two times incubation for 10 minutes in LiChrosolv ethanol (Merck) and then three times for 5 minutes in PBS.

### Fluorescence *in situ* hybridisation

FISH was performed on previously fixed and embedded tissue sections. The fixed sections were covered with hybridisation buffer (900 mM NaCl, 20 mM Tris–HCl, 0.01% SDS, 25% formamide) which was premixed with four different probes (each at 5 pM) in a 1:10 (probe to buffer) ratio and hybridized at 46°C for 2 h. Probes used for hybridisation included a mix of Chls-523 (5’-Cy3 CTTCCGTATTACCGCAGC -3′) [25] and Chls-282 (5’-Cy3 CTCAATCCGCCTAGACGT -3′) [26] for specific detection of chlamydiae, a mix of EUB338 I (5′-6-FAM GCTGCCTCCCGTAGGAGT 6-FAM-3′), EUB338 II (5′- 6-FAM GCAGCCACCCGTAGGTGT 6-FAM-3′), EUB338 III (5′-6-FAM GCTGCCACCCGTAGGTGT 6-FAM-3′) [27] for detection of bacteria, and as control for unspecific probe binding the NonEUB probe (5’-Cy5 ACTCCTACGGGAGGCAGC -3′) [28]. Slides were washed for 15 minutes in washing buffer (20 mM Tris–HCl, 0.25 mM EDTA and 0.149 M NaCl) at 48°C and briefly dipped in ice-cold deionized water. Slides were then stained with 4′,6-diamidino-2-phenylindole (DAPI) for 7 minutes and washed in 99% ethanol for 1 minute. Imaging was done on a TCS SP8 X confocal laser scanning microscope (Leica Microsystems) equipped with a 93x glycerol objective and the Thunder Imager Live Cell and 3D Imager (Leica Microsystems) with a 63x glycerol-immersion objective and a 100x oil objective. Images were analyzed using the Leica application suite X software v3.7.6 (Leica Microsystems).

### Transmission electron microscopy (TEM)

Two individuals of symbiotic *H. oligactis* Montaud polyps, were maintained without food for 2 days. Then individuals were transferred into high-pressure freezing type A carriers (3 mm diameter, 200 μm depth) and covered with 10% bovine serum albumin and type B carriers (Leica Microsystems). Before sample application the carriers were coated with 1-hexadecene. After closing, the samples were frozen at ~2000 bar, using an HPM100 high-pressure freezer (Leica Microsystems). Frozen samples were freeze substituted in an automated freeze substitution system AFS2 (Leica Microsystems). For this, carriers containing high-pressure frozen samples were placed in −90°C 1% OsO_4_ in acetone in 2 ml cryotubes. Freeze substitution was performed under agitation following this temperature program: 2 h from −105 to −95°C, 40 h at −95°C, 2 h from −95°C to −90°C, 5 h at −90°C, 2 h from −90°C to −60°C, 2 h at −60°C, 4 h from −60°C to 20°C. Then samples were washed with acetone and infiltrated with epoxy resin Epon (Agar100 Scientific Ltd.) at room temperature. Polymerization of the resin was conducted at 63°C for 72 h. Ultrathin sections of 70 nm thickness were prepared using a diamond knife (Diatome) with a Leica ultramicrotome EM UC7 (Leica Microsystems) and mounted on 200 mesh copper grids. Contrasting was done using 4% Neodymium(III) acetate hydrate (Thermo Fisher Scientific) for 50 minutes and 3% lead citrate for 8 minutes. The stained ultrathin sections were examined with a Libra 120 transmission electron microscope (Zeiss) with a Bottom mount camera Sharp:eye TRS (2 × 2 k).

### Isolation and cultivation in insect cells

Insect cells (*Spodoptera frugiperda* Sf9 cell line) were cultured in Grace’s insect medium (Thermo Fischer Scientific) supplemented with 10% fetal bovine serum (Thermo Fischer Scientific) at 28°C. Chlamydial EBs were harvested by transferring >200 *Hydra* polyps into a 1 ml Wheaton Dounce tissue homogenizer and douncing 40 times with the loose pestle and 40 times with the tight pestle. Sufficient lysis of the cells was ensured using epifluorescence microscopy on DAPI stained lysates, using a Zeiss Axio imager A1 microscope. Bacterial cells were then filtered using a 1.2 μm filter. EB density was assessed with the QUANTOM Tx Microbial Cell Counter (Logos biosystems). Insect cells were seeded in a 6-well plate (Thermo Fischer) at a density of 1 × 10^5^ cells/ml and infected with EBs at a multiplicity of infection of 100 or higher. To prevent contamination 10 μl/ml of 100x antibiotic, antimycotic solution (Sigma-Aldrich) were added. To enhance infection probability the plate was centrifuged at 500 × g for 15 minutes and incubated at 28°C. Insect cells were grown until confluence and transferred into 75 cm^2^ cell culture flasks (Nunc EasYFlask). Infection success was monitored monthly with PCR of 1.2 μm filtered culture supernatant, using the chlamydiae-specific primer pair mentioned above and subsequent Sanger sequencing (Microsynth AG). After five months of co-cultivation, FISH was performed as described above.

### 16S rRNA gene-based diversity and environmental distribution of *E. Hydrae*

The integrated microbial next-generation sequencing (IMNGS) database, which is a collection of pre-clustered NCBI SRA sequencing data [29], was queried on 25 November 2024 for 16S rRNA genes with at least 95% identity to the reference 16S rRNA gene sequence of *E. hydrae*. The categories provided by the SRA were manually curated so that sequences were assigned to one of the following categories: freshwater, freshwater sediment, groundwater, marine, marine sediment, soil, and wastewater (Supplementary Table S3).

### Sampling, sequencing, and analysis of wild *Hydra*-associated chlamydiae

Three distinct *H. oligactis* populations were sampled in Hungary over a period of two years (between June 2021 and June 2023). Each population was sampled once every season. At each sampling three *Hydra* polyps and one water sample were collected from three distinct locations along shorelines and brought to the laboratory on the day of collection. Polyps were washed with sterile-filtered water to remove debris, and water was sterile-filtered to obtain DNA samples from bacterioplankton (no water samples were available for the first sampling event in June 2021). DNA extraction was performed with a phenol-chloroform extraction method as previously described [30] and extracted DNA was sent to Novogene (Cambridge) for amplicon sequencing of the V4 region. *Uparse* v7.0.1001 was used to cluster OTUs based on the SILVA database (version 138.1) with a 97% similarity threshold. Detailed methods for sampling and sequencing have been described previously [31].

OTUs belonging to the phylum *Chlamydiota* were extracted using the R package phyloseq [32]. Relative abundances between chlamydiae in each sample were calculated and visualized in a heatmap using R version 4.4.2 and the package pheatmap. Chlamydial sequences were further processed by calculating 16S rRNA gene phylogeny with other members of the *Chlamydiota*.

### 16S rRNA gene phylogeny of lab and wild derived strains

A dataset of 481 nearly full length (>1200 nt) 16S rRNA gene sequences was generated. This contained chlamydial sequences derived from a previously published dataset ([33], n = 55), genomes available on NCBI on 11 April 2024 (n = 42), as well as 245 chlamydial and 139 outgroup sequences belonging to the *Planctomycetota*, *Omnitrophota*, *Verrucomicrobiota*, and LD1-PA32 phyla [34]. Full length sequences not yet aligned to the SILVA alignment, were aligned with MAFFT v7.490 (−-add --keeplength) [35]. The alignment was trimmed with trimAl v1.5.0 (−-gappyout) [36]. A reference tree containing the full length 16S rRNA gene sequences was generated with IQ-TREE v2.3.5 using the SYM + R10 model with 1000 ultrafast bootstraps and the SH-like approximate likelihood ratio test [37, 38]. Partial 16S rRNA gene sequences from the environmental screening were added to this alignment with MAFFT v7.490 (−addfragments –keeplength) [35], subsequently added to the full length reference tree with EPA-ng v0.3.8 (−m GTR + G) [39], and visualized in iTOL v7.0 [40].

### Sequencing and assembly of the genome

200 or 115 individuals of the symbiotic *H. oligactis* strain Montaud were maintained without food for 2 days for high or low molecular weight DNA isolation, respectively. Then symbiont cells were harvested as described above. The remaining cell lysate was filtered consecutively through 5 μm and 1.2 μm filters (Sartorius). The filtrate was analysed for sufficient removal of host cells, using epifluorescence microscopy (as described above). Bacteria were harvested by centrifugation at 15 500 × g for 15 minutes at 4°C. The remaining cell pellet was resuspended in PBS and immediately used for extraction of genomic DNA.

Extraction of high molecular weight DNA was carried out using the Wizard HMW Extraction kit (Promega) according to the instruction manual, with few deviations. In brief, all centrifugation steps were performed at 15 500 × g, drying of the DNA pellet was done at 60°C for 5 minutes and the subsequent rehydration of the DNA was carried out overnight at room temperature in 25 μl of rehydration solution. Library preparation was performed at the Vienna Bio Center Core Facilities using the Multiplex Ligation Sequencing Kit and the Native Barcoding Kit (Oxford Nanopore Technologies). Long-read sequencing was performed on an ONT Flongle system. Extraction of low molecular weight DNA was done using the DNeasy Blood & Tissue Kit (Qiagen). Libraries for short read sequencing were prepared at the Joint Microbiome Facility (University of Vienna and Medical University of Vienna) using the NEBNext Ultra II FS DNA Library Prep Kit for Illumina (New England Biolabs), pooled equimolarly, and sequenced on a MiSeq System (Illumina) (2x 300 bp, 600 cycles). Short reads were trimmed using BBDuk (part of BBMap v39.01 [41]) to a minimum of 50 base pairs in length to avoid adapter contamination and low quality sequences. Long reads were trimmed using Porechop 0.2.4 [42]. In total we obtained 1 156 262 short and 112 117 long reads of which roughly 43% and 11% respectively were assembled into a circular genome using Unicycler v0.5.0. [43]. The result was a single chlamydial contig and the origin of replication was determined using Ori-finder 2022 [44].

### Phylogenetic analysis

Average nucleotide identity (ANI) was calculated using FastANI v1.34 [45] against a set of 226 chlamydial genomes (Supplementary Table S4), resulting in no hits against *E. hydrae*. Then a concatenated alignment (Supplementary Data S1) of 43 CheckM marker proteins (Supplementary Data S2) from 226 chlamydial genomes and 90 *Planctomycetota*-*Verrucomicrobiota* outgroup representatives was generated using CheckM v1.2.3 [46]. This alignment was then used to infer a maximum likelihood phylogenetic tree using IQ-TREE v.2.2.6. [37] with the model LG + C20 + R4 as selected by Modelfinder plus [47]. The final tree was calculated using 1000 ultrafast bootstraps repeats [38] and 1000 replicates of the SH-like approximate likelihood ratio test [48], it was rooted using the outgroup and visualized with iTOL v.7.0 [40].

### Annotation and comparative genomics

Gene calling and annotation were performed using bakta v1.9.3 [49] and eggNOG-mapper v2.1.11 [50]. We analysed metabolic pathways, transporters, and secretion systems using KEGG [51], in combination with InterProScan 5 [52]. The presence of specific genes of interest (anaerobic pathway genes, virulence genes, T3SS) were additionally investigated using BLAST and known sequences from the complete, high-quality genomes of either *Simkania negevensis* or *Chlamydia trachomatis*. Secondary metabolites were predicted using antiSMASH v8.0.4 [53].

A comprehensive dataset of all encoded proteins in 226 closed chlamydial genomes and high-quality MAGs (Supplementary Table S4) were clustered into orthologous groups (OGs) using OrthoFinder v2.5.5 [54] under default parameters. Selected OGs that did not show any sequence similarity to known proteins in public databases were used for structure prediction using AlphaFold v.2.3.2 [55] and compared with structures in the RCSB protein data bank (PDB) [56].

Genomic islands (GIs) were predicted using Islandviewer4 based on Islandpath-DIMOB which is integrating multiple features such as GC bias*,* dinucleotide bias*,* the presence of tDNAs, and mobility-related genes [57]. GC skew was calculated using a custom R script (window size = 1000). Transposases were identified in two parts, by extracting all sequences annotated as transposases in the bakta annotation file (Supplementary Table S5) together with sequences from two Orthofinder OGs (OG0001333, OG0000802) identified as insertion sequences. To identify larger insertion sequences around the transposon sequences, they were aligned using Aliview [58], grouped based on similarity and further aligned with the *E. hydrae* genome using Unipro UGENE [59]. To identify genes under degradation in the genome of *E. hydrae* we used pseudofinder (v1.0) [60] and compared the genome against a database comprising 25 other *Endochlamydiaceae* genomes (Supplementary Table S4) using a threshold of 0.3 to distinguish between pseudogenes (>0.3) and genes under purifying selection (<=0.3). GC skew, genomic islands, IS sequences and pseudogenes were visualized using the R package circlize [61].

## Results and discussion

### Stable association of a chlamydial symbiont with *H. oligactis*

**Figure 1:**
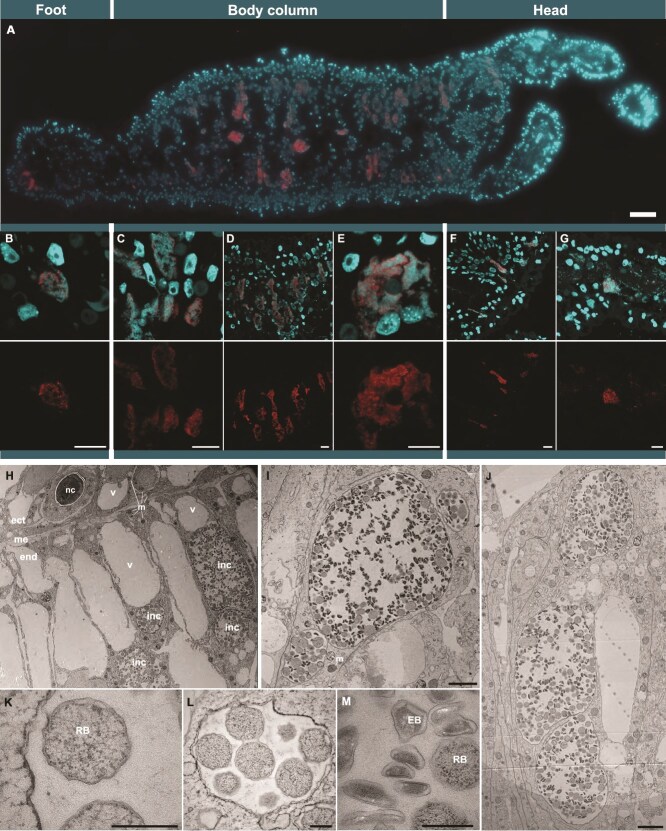
Chlamydiae infect the endoderm of *Hydra oligactis*. (**A**) Chlamydiae are located inside the endoderm along all parts of the *H. oligactis* polyp, as observed by FISH and confocal laser scanning microscopy on 4 μm sections of three different individuals of *H. oligactis* strain Montaud. (**B–G**) Zoom in on different areas (foot, body column, head + tentacles) showing chlamydiae inside the endoderm of polyps with FISH and microscopy. Red: Chlamydiae-specific oligonucleotide probes Chls523 and Chls282 (chlamydial probe mix); cyan: DNA (DAPI staining). Images in the upper panel show overlay of the chlamydial probe mix and DAPI staining, images in the lower panel show the signals of the chlamydial probe mix. (**H–M**) transmission electron micrographs showing large inclusions within the *Hydra* endodermal cells (H–J). Close-ups of the chlamydial reticulate bodies in small inclusions (K, L) and of elementary bodies in a large inclusion (M). Ectoderm (ect), mesoglea (me), endoderm (end), nematocyst (nc), vacuole (v), mitochondria (m), chlamydial inclusion (inc), chlamydial reticulate bodies (RB), chlamydial elementary bodies (EB). Scale bars: 50 μm (A), 10 μm (B–G), 2.5 μm (H–J), and 0.5 μm (K–M).

**Figure 2:**
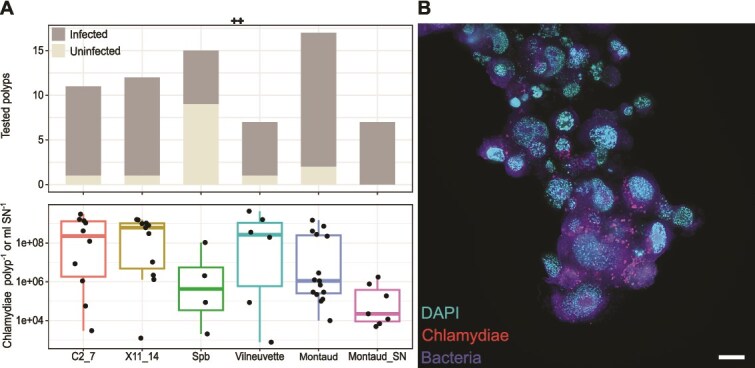
Abundance of chlamydiae in *Hydra* lab strains and stable reproduction in an insect cell line. (**A**) Number of tested positive (grey) and negative (ie, uninfected; light-grey) individuals of each *H. oligactis* strain are shown in the bar plot. Below, total chlamydial abundance in individual polyps of the same *H. oligactis* strains or culture supernatant (SN) as measured by dPCR. Boxes indicate the interquartile range, the horizontal line indicates the median, whiskers indicate minimum and maximum values, respectively. (**B**) Chlamydiae are located within Sf9 cells, observed after five months of co-cultivation with FISH and thunder imager. Cyan: DNA stain DAPI; red: Chlamydiae mix (probes Chls523 and Chls282), blue: Eubacteria mix (probes EUBI + EUBII + EUBIII). Scalebar 10 μm.

A previous study suggested that a single chlamydial organism represents a large fraction of the microbiome of *H. oligactis* laboratory cultures established between 2016 and 2021 originating from environmental samples [23]. To confirm these observations and to investigate the stability of the association between *Hydra* and this chlamydial symbiont, individuals of the symbiotic *H. oligactis* strains SpB, X11/14, C2/7, Vilneuvette, and Montaud [23, 62] were screened with PCR. Additionally, individuals of the strain Montaud were monitored in culture regularly by FISH of sectioned individuals and dPCR of both individuals and culture supernatant. The chlamydial symbionts were detected consistently within the tissue of the *Hydra* strain Montaud by FISH throughout a 2-year cultivation period (Fig. 1). 16S rRNA gene sequencing and dPCR using DNA isolated from individual *Hydra* polyps of all strains allowed us to verify the symbiont identity and quantify the number of chlamydiae present per animal. Polyps of each strain testing negative or positive for chlamydiae were kept in the same culture vessels until analysis. Of the 62 analyzed *H. oligactis* individuals from all strains mentioned above 77.4% were, at the time of harvesting, infected with chlamydiae (Supplementary Table S1). On average a single infected polyp of *H. oligactis* strain C2/7, Montaud, Spb, Vilneuvette, and X11/14 harbored 7.8 × 10^8^, 2.1 × 10^8^, 2 × 10^7^, 1.1 × 10^9^, and 6.4 × 10^8^ chlamydiae, respectively (Fig. 2A). Variance however was high between tested individuals, ranging from 766 up to 4.4 × 10^9^ chlamydiae per polyp (Fig. 2A, Supplementary Table S1). Further analysis was restricted to *H. oligactis* strain Montaud and further monitoring of chlamydial presence in animals was based on microscopic observations.

### Chlamydiae reside in large inclusions within the endoderm of the *Hydra* polyp

To visualize the location of chlamydiae within *Hydra* polyps, we used FISH with chlamydiae-specific probes on thin sections of whole animals. We observed chlamydiae-containing inclusions exclusively associating with epithelial cells of the *Hydra* endoderm along the entire body column of the animal, including foot and tentacles (Fig. 1A–G). Subsequently we confirmed the location of chlamydial cells inside the *Hydra* endodermal cells, appearing exclusively in densely filled inclusions with lengths of up to 25 μm, by TEM (Fig. 1H–M). We identified morphotypes characteristic of the chlamydial developmental stages: EBs were often elongated, with 300–520 nm in length, and located in larger inclusions more frequently in the center of inclusions (Fig. 1H–J). RBs appeared round, with 480–860 nm in diameter (Fig. 1K–M), and were more prevalent at the periphery of inclusions (Fig. 1H–J). This is consistent with observations of other chlamydiae that form large inclusions like the human pathogen *C. trachomatis* [63] and the amoeba symbiont *Parachlamydia acanthamoebae* [64].

### Evidence for horizontal and vertical transmission

Chlamydial EBs enter a host cell via a combination of phagocytosis and chlamydiae-induced actin remodeling at the entry site [63]. After entry and transformation to replicating RBs, they eventually transform back to EBs that are typically released via host cell lysis or inclusion extrusion [65–67], sometimes also through cell-to-cell contact [68, 69]. In this *Hydra*-chlamydiae system we could not directly observe evidence for lysis or chlamydial extrusion in *Hydra* endodermal cells in TEM.

We tested for the presence of chlamydial DNA in the supernatant of *H. oligactis* strain Montaud cultures, using dPCR, and found on average 3.9 × 10^5^ chlamydial genome copies/ml in the supernatant of all tested cultures (n = 7), whereas infected polyps of the same culture had on average 7.8 × 10^8^ copies/polyp (Fig. 2A, Supplementary Tables S1 and S2). The detection of chlamydial genome copies in the supernatant is not a direct proof for the presence of infectious EBs and horizontal transmission between individual animals; it could also result from residual DNA in the supernatant. For *C. trachomatis* it has been shown that infectious EBs in cervical swabs differ by a factor of 257 (ranging from 4 to 2035) from genome copies [70], suggesting that at least a fraction of the genome copies detected in the supernatant of *Hydra* cultures represents viable chlamydial EBs.

Because all *H. oligactis* Montaud cultures in our lab were infected with chlamydiae, and we were not able to obtain uninfected individuals of this strain, we further studied horizontal transmission of chlamydiae from *H. oligactis* by establishing infection in Sf9 insect cells, which have been shown previously to be susceptible to chlamydial infection [4, 71]. Using chlamydiae-specific PCR and FISH, we could show that the chlamydial symbionts could stably infect Sf9 cell cultures (Fig. 2B).

**Figure 3:**
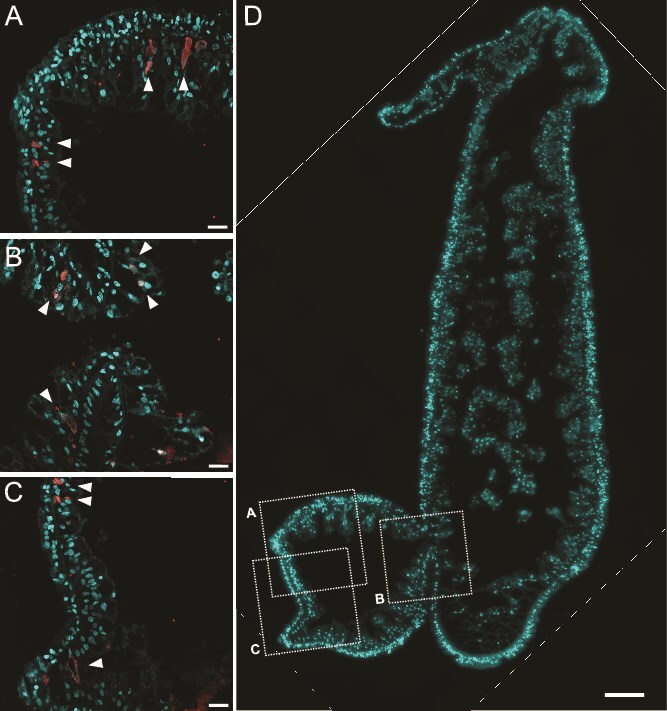
Symbionts are transmitted vertically through the endoderm. (**A–C**) Chlamydiae-containing inclusions appear throughout the endoderm of forming *Hydra oligactis* buds, as observed with FISH and confocal laser scanning microscopy. (**D**) Overview of the section with DAPI, as observed with FISH and thunder imager. Red: Mix of Chls523 and Chls282 probes; cyan: DAPI stain. Scale bars 20 μm (A–C) and 100 μm (D).

When sufficient food is available *Hydra* mainly reproduces asexually by budding. In *H. oligactis* this mode is preferentially used over sexual reproduction, which especially in females typically results in the death of the polyp [72]. We were able to confirm vertical transmission of chlamydial symbionts to the offspring during asexual reproduction, using FISH on thin sections of *H. oligactis* Montaud polyps (Fig. 3).

Taken together, chlamydial DNA potentially representing EBs is found in the culture supernatant of infected *H. oligactis* polyps. Collected chlamydiae from these cultures can be propagated in insect cell cultures, suggesting horizontal transmission via the environment. In addition, chlamydial symbionts are transmitted vertically to newly forming buds during asexual reproduction of the *Hydra* polyp. Albeit this ensures propagation and spread of the symbiont within the host population, vertical transmission generally stabilizes host-symbiont associations and has been shown to create a coupling of host- and symbiont fitness, leading to decreased virulence [73].

### Potential environmental reservoir

**Figure 4:**
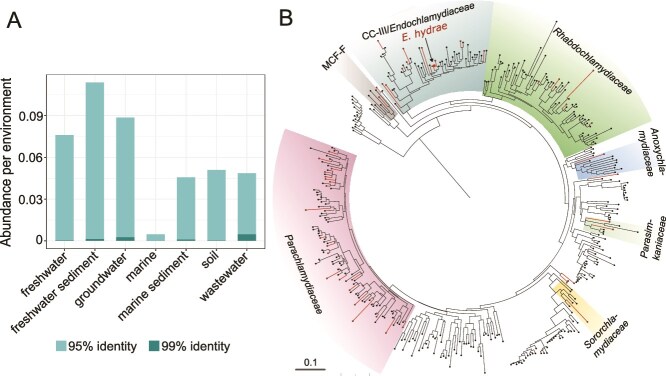
Diversity and distribution of *Hydra*-associated chlamydiae in the environment. (**A**) Occurrence of *E. hydrae* 16S rRNA gene sequence in microbiomes of selected environments at the species (99% identity) and genus (95% identity) levels. Abundance is shown as the percentage of microbiomes within the IMNGS database in which the sequence could be found at either identity level. (**B**) Maximum likelihood phylogeny of 16S rRNA gene (V4 region) of 51 chlamydial genus-level OTUs. Red colored branches represent taxa from which a chlamydial sequence originating from a *Hydra* polyp could be detected. The *E. hydrae* phylotype is labeled additionally.

To assess the environmental distribution of the *Hydra*-associated chlamydial symbiont, we searched available microbiome data sets for similar 16S rRNA gene sequences using the IMNGS database [29]. On the species level (99% sequence identity with the 16S rRNA gene of the symbiont), environments with highest relative abundances were found in wastewater, groundwater, and freshwater sediment microbiomes. On the genus level (95% sequence identity), highest relative abundances were found in freshwater sediment (0.11%), groundwater (0.09%), and freshwater (0.08%) (Fig. 4A, Supplementary Table S3). The detection of 16S rRNA gene sequences similar to that of the *Hydra* symbiont at the species and genus-level in environments with low expected *Hydra* abundances such as groundwater and wastewater, suggests that their broad distribution might be facilitated by other hosts. Small invertebrates, cnidarians, and many protists, common hosts of chlamydiae [3], thrive in these environments, and some protists like the ciliate *Kerona pediculus* are known to colonize the surface of *Hydra* polyps [74].

### Diverse chlamydiae associated with wild *Hydra* populations

Little is known about the association of chlamydial symbionts with wild *Hydra* populations. We thus analysed chlamydial 16S rRNA gene sequences from a microbiome dataset of wild *H. oligactis* populations and surrounding environmental water samples [31]. Chlamydial sequences identical or similar (>95% sequence identity) to the chlamydial symbiont of the lab strains were present in several wild *Hydra* polyps. Chlamydial sequences detected here were not only restricted to the *H. oligactis* symbiont phylotype but were highly diverse and affiliated with several chlamydial families, including the *Parachlamydiaceae* (24 operational taxonomic units, OTUs), *Rhabdochlamydiaceae* (5 OTUs), and CC-III/*Endochlamydiaceae* (12 OTUs) (Fig. 4B). Most sequences, including the *H. oligactis* symbiont phylotype, were detected both in polyp and water samples (Supplementary Fig. S1, Supplementary Table S6, Supplementary Data S3).

The relative abundance of chlamydial sequences in the environmental polyps was very low (<0.0001% in most samples, with a maximum of 0.04%) [31]. This might represent an underestimate due to known biases of commonly used bacterial primers discriminating against the *Chlamydiota* [75, 76] and contrasts with the high (>50%) relative abundance found in *H. oligactis* Montaud lab populations [23]. The presence of chlamydial sequences in the samples from wild *Hydra* populations, however, could either represent *Hydra* symbionts or chlamydiae present in the water or in microbial eukaryotes that were not efficiently removed during sample preparation. Together, the detection of chlamydial DNA in wild *Hydra* populations identical to the symbiont of *H. oligactis* Montaud lab strains strongly suggests the presence of the symbiont in natural environments. In addition, there is evidence for an even higher diversity of chlamydiae associated with wild *Hydra* populations and their microbiomes.

### *Endochlamydia hydrae* a cultured representative of the *Endochlamydiaceae* (CC-III) family

**Figure 5:**
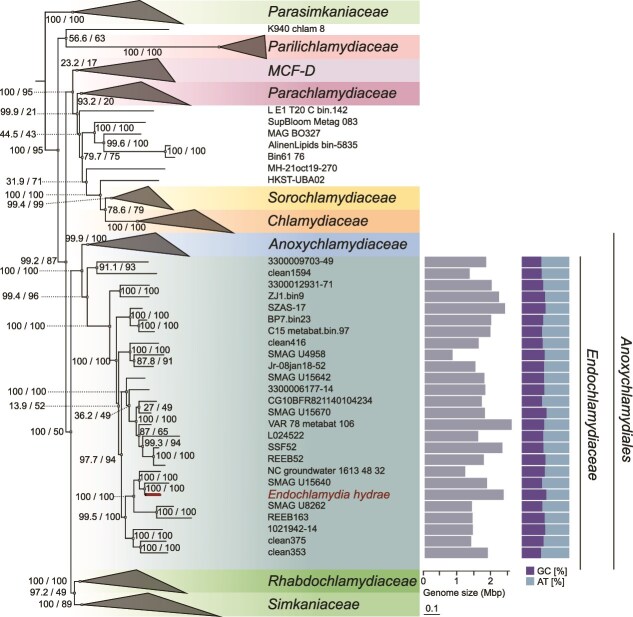
Phylogenetic tree showing the relationship of the *H. oligactis* symbiont *Endochlamydia hydrae* in the phylum *Chlamydiota.* The symbiont represents an isolate of the family *Endochlamydiaceae* (formerly CC-III) within the order *Anoxychlamydiales*, that includes the two families *Endochlamydiaceae* and *Anoxychlamydiaceae*. A maximum likelihood phylogenetic tree based on 43 concatenated conserved marker proteins in 226 chlamydial genomes is shown. Support values based on 1000 ultrafast bootstrap replicates and 1000 replicates of the SH-like approximate likelihood ratio test are indicated on the branches. Scale bar represents 0.1 amino acid substitutions per site. Bars indicate size (left) and percent GC and AT content (right) of *Endochlamydiaceae* genomes.

We obtained the full genome sequence of the chlamydial symbiont of *H. oligactis* through a combination of long and short read sequencing. With a size of around 2.3 Mbp and 2542 coding sequences the genome is larger than those of chlamydial vertebrate pathogens but similar in size to those of protist-associated chlamydiae. The GC content of 51.4% is at the upper limit in the phylum [1, 77] (Supplementary Table S4). Based on phylogenetic analysis and average amino acid identity (Supplementary Fig. S2) comparison this symbiont represents an undescribed species within the recently proposed CC-III family (Fig. 5) [1]. A family, so far described only based on metagenome-assembled genomes (MAGs) from environments including freshwater, groundwater, and marine seawater [1]. The *Hydra* symbiont is to our knowledge the only cultured representative of this family. For this species we propose the name *Endochlamydia hydrae*, and consequently for the CC-III family we propose the name *Endochlamydiaceae* (for detailed descriptions see Supplementary Text)*.*

The genome of *E. hydrae* contains most of the well conserved hallmark genes typically found in the *Chlamydiota* (Supplementary Table S5) [1]. It encodes genes and complete pathways for energy parasitism (nucleotide scavenging genes *tlcA-C*) [13, 78], virulence genes like the T3SS, many adhesin proteins (n = 13), and several serine–threonine kinases (*pknD*, *pkn1*, *prkC*, RIPK4) (Supplementary Table S5) [77].

The genome contains full pathways for menaquinone synthesis and shikimate, enabling the synthesis of aromatic amino acids. Genes for the production of secondary metabolites are usually organized in biosynthetic gene clusters (BGCs) [79] and have been found to be widespread across the *Chlamydiota* [80]. In *E. hydrae* five such BGCs were predicted. They include a cluster for Nonribosomal Peptide Synthetase–like proteins [81], a cluster for the production of proteusins [82], type III polyketide synthases [83], and two terpene precursor clusters (Supplementary Table S7), which all potentially offer defense against other microbes infecting *Hydra* or other hosts by the production of anti-microbial substances.

*E. hydrae* is capable of acetogenesis by the oxidative carboxylation of pyruvate to acetate and carbon dioxide, using the pyruvate:ubiquinone oxidoreductase. In addition, we found a complete de novo pyrimidine synthesis pathway, a feature present only in two other environmental chlamydiae that would enable greater independence from any host [13, 84, 85]*.*

### Genome compositional dynamics of *E. hydrae*

**Figure 6:**
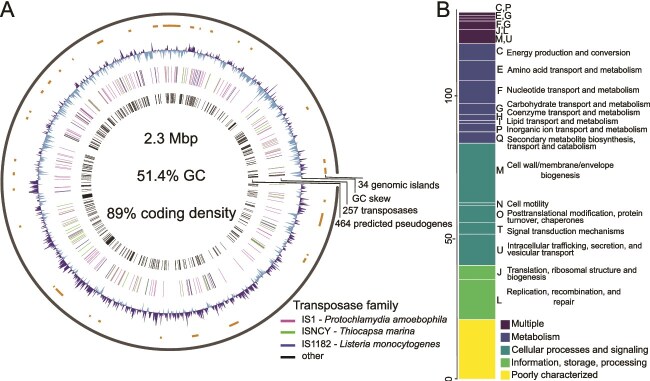
The genome of *E. hydrae* shows signs of many genomic disruptions and rearrangements. (**A**) Circular plot of the *E. hydrae* genome. The outermost ring shows genomic islands (orange), predicted by IslandPath-DIMOB using Islandviewer4. The second ring shows positive (darkblue) and negative (lightblue) GC skew. The third ring represents both putative and functional transposase genes. Transposases are coloured according to sequence similarity to IS families. The innermost ring shows pseudogenes present in *E. hydrae* compared to other *Endochlamydiaceae* genomes, predicted using pseudofinder. Legend shows transposases listed from highest to lowest abundant sequence together with their closest ISfinder BLAST hit. (**B**) Functional categories of predicted pseudogenes with a COG annotation in *E. hydrae*.

The *E. hydrae* genome size of 2.3 Mbp supersedes that of other family members and with 2542 predicted coding sequences possesses a high coding density of 89.4%. In addition, we identified a large number of pseudogenes (n = 464) compared to other *Endochlamydiaceae* genomes, representing ~18% of the total number of predicted coding sequences (Fig. 6A). These genes fall into a wide spectrum of functional categories according to clusters of orthologous gene (COG) classification (Fig. 6B, Supplementary Table S8). Although studies regarding pseudogenes in chlamydiae are scarce, reported numbers for *Chlamydiaceae* have been low to none [86].

We focused on transposase genes as potential causative agents for pseudogene formation and genome streamlining. Transposases are indicative of transposable elements, that in the simplest form of insertion sequences consist of only a transposase gene flanked by inverted repeats. They can spread through a genome, disrupting and rearranging genomic content [87]. In endosymbionts they have been shown as mediating genome size reduction under relaxed selection, leading to accumulation of mutations and inactivation of genes [88–90]. A comparison of two closely related *Rhabdochlamydia* species in presumed early and late stages of host transition, has found a high number of transposase genes and pseudogenes in the more recently transitioned species [4]. In the genome of *E. hydrae,* we find a high number of IS elements (n = 257) similarly spread across the entire genome (Fig. 6A). They are dominated by three distinct transposase genes belonging to three different IS element families (Supplementary Table S9). The most abundant transposase genes belong to the IS1 element family, which make up over 60% of all transposases in the genome and were found to be highly conserved (Supplementary Table S9). This suggests a relatively recent proliferation of IS1 elements in the *E. hydrae* genome, whereas the other, more degraded IS element families could represent remnants of previous IS element radiation events.

Another feature of the *E. hydrae* genome is an unusually large number of predicted GIs, ie, genomic regions that deviate in nucleotide composition and are typically indicative of horizontal gene transfer events (Fig. 6A). Based on their gene content and the sequence similarity to other chlamydiae (eg, many ribosomal proteins, Supplementary Table S10) the predicted GIs likely do not represent horizontal transmission events but are rather consequences of genome reorganization and remodeling events. This scenario is consistent with the observed pattern of the cumulative GC skew, which showed a less pronounced trend in *E. hydrae* (Fig. 6A) than the characteristic asymmetric pattern usually found in circular chromosomes [91].

Because of the unusual high genomic GC content of *E. hydrae* of 51.4% compared to around 42.6% in most other chlamydiae ([77], Supplementary Table S4), we further studied GC dynamics across the *E. hydrae* genome (Supplementary Fig. S3, Supplementary Table S11). Intergenic regions showed a GC content of 43.1%, whereas the GC content in coding genes is with 52.4% similar to the genomic GC content. An even higher GC content was observed at the third codon position, with GC3 = 60.9%. The GC content in transposase-encoding genes did not differ markedly from those of other genes. The amplification of transposase genes is thus not the cause of the high GC content in *E. hydrae* (Supplementary Fig. S3). Rapid changes, termed “evolutionary jumps”, in GC content have been reported for other bacterial lineages, putatively driven by ecological and evolutionary factors [92], and selection might particularly favor an increase in GC3 [93].

Taken together, the high proportion of pseudogenes, the abundance of highly conserved IS elements, the irregular GC skew, and the high GC3 content are indicative of evolutionarily recent or still ongoing genomic rearrangements and genome transformation in *E. hydrae* and point to adaptations to *Hydra* as a relatively new host or even recurring switches between *Hydra* and/or other hosts. In addition *E. hydrae* forms a rather short branch in the *Endochlamydiaceae,* with a relative evolutionary divergence [94] of 0.022 to its nearest neighbour and 0.045 to the nearest branch outside the genus (Fig. 5, Supplementary Fig. S4).

### Potential host-symbiont interaction features

Overall, *Chlamydiota* genomes have a comparatively large core genome, a limited accessory genome with genes shared by different subgroups of organisms, and few unique gene families [1, 77]. We analysed species-specific genes by comparing OGs of *E. hydrae* and 225 other chlamydial genomes (Supplementary Table S4). A total of 254 genes clustered into 19 different OGs were found to be unique to *E. hydrae* (Supplementary Table S12). 211 of those genes encode transposases, later identified as part of IS elements described above. Other unique genes contain signal peptide and membrane protein domains, none of which could be assigned a specific function (Supplementary Table S12).

Among the unique genes were also two genes encoding ankyrin repeat domains (ANK). ANK domains are typically found in a class of eukaryotic-like proteins (ELPs) that are widespread in bacteria, mediating protein–protein and protein-nucleic acid interactions [95]. They are regarded to have originated via horizontal gene transfer from eukaryotes to bacteria [96] and are considered to be part of a system ensuring bacterial survival in host cells [97]. Other ELPs encoded in the genome of *E. hydrae* include proteins with tetratricopeptide repeats (TPR, n = 12), membrane occupation recognition nexus repeat proteins (MORN, n = 4), and WD40 repeat proteins (WDR, n = 2) (Supplementary Table S13). Taken together, the unique genes and ELPs found in *E. hydrae* could represent small-scale adaptations to the *Hydra* host.

When extending our analysis to the entire *Endochlamydiaceae* family, we were able to identify seven OGs that are unique to the family and are present in at least 15 out of 24 *Endochlamydiaceae* genomes. Two OGs were detected in 23 out of 24 genomes (Supplementary Table S12). OG0002141 consists of a protein with a signal peptide, the other OG0002142 has no homologs to proteins in the NCBI or Interpro databases. Predictions of the structure using AlphaFold2 [55] did not provide conclusive results about the function of this protein. Similarly only a small number of species or family specific genes have been observed for chlamydiae infecting sponges and corals [5, 80]. Thus, our findings further support the notion that chlamydiae are in general well adapted to infect eukaryotic hosts and only need a limited number of additional adaptations for interacting with non-chordate host organisms.

### Model for studying adaptations to host innate immune response

*Hydra* belongs to one of the basal metazoan lineages and possesses only a basic innate immune system with TLR-like and NLR-like pattern recognition systems that are linked to a basic NF-kB pathway and the activation of antimicrobial peptide production to control bacteria [19–21]. The symbiosis with *E. hydrae* offers a bridge between chlamydial hosts without an immune system—such as protists—towards chordates with innate and adaptive immune responses. Of the various genes characterized in chordate-infecting chlamydiae that interfere with the host innate immune system only the tail-specific protease (Tsp, CT441) is encoded in *E. hydrae* (HYCHLAM_01890, HYCHLAM_12045, Supplementary Table S5). Tsp blocks IκBα degradation and thus the activation of NF-kB and as a consequence antibacterial response [98, 99]. Tsp containing the PDZ domain responsible for this activity is one of the chlamydial core genes present in all members of the phylum that might be able to interfere with the basic innate immune system of *Hydra*.

## Conclusion

In this study we characterized a cultivated isolate of a member of the family *Endochlamydiaceae* as an endosymbiont in the endoderm of the freshwater polyp *H. oligactis*. An in-depth characterization of the *E. hydrae* genome revealed that it encodes all the hallmark genes characteristic for the chlamydial intracellular lifestyle and showed only a small set of unique genes, some of which potentially play a role in host interaction and may represent specific adaptations to *Hydra* as a host. Similar to *E. hydrae*, other chlamydial families prevalent in marine cnidarians or sponges, were described as having little genomic specialization, suggesting a lack of host specificity among those chlamydiae [5, 80]. We have instead found evidence for ongoing genomic rearrangements and interpreted this to be the result of a more recent or constant host switch and ongoing host adaptations.

*Hydra* represents yet another early eukaryotic lineage serving as natural host for chlamydiae, which further underscores the versatility and success of this bacterial phylum to infect an immensely diverse range of eukaryotes. The availability of an *E. hydrae* culture as a representative of a distinct and phylogenetically diverse chlamydial family offers the potential to study the evolution of host interaction and adaptation, as well as adaptations to the *Hydra* immune response. In the future this might contribute to a better understanding of the evolution of important human and animal pathogens in the phylum.

## Supplementary Material

Supplementary-Material_wrag104

## Data Availability

The sequencing data for the field collected *Hydra-*associated chlamydiae are available under PRJNA1119407; the assembled genome sequence and sequencing data of *E. hydrae* are available under PRJEB86254.
