# Supplementary material for: Stable association of a chlamydial symbiont with the freshwater predator hydra suggests broad host potential
Source: ISME J. 2026 Apr 30;20(1):wrag104. doi: 10.1093/ismejo/wrag104 (PMC13229535; doi:10.1093/ismejo/wrag104)
Supplement: Supplementary-Material_wrag104 [file supplementary-material_wrag104.zip › Supplementary_information_wrag104.pdf]

Stable association of a chlamydial symbiont with the freshwater predator *Hydra* suggests broad host potential

[1,2]Angelika Schwarzhans, [3]Justine Boutry, [4]Jácint Tökölyi, [5]Norbert Cyran, [1]Martin Kunert, [1]Matthias Horn, [1]Astrid Collingro

[1]Centre for Microbiology and Environmental Systems Science,
University of Vienna, Djerassiplatz 1, 1030, Vienna, Austria

[2]Doctoral School in Microbiology and Environmental Science, University of Vienna, Djerassiplatz 1, 1030 Vienna, Austria

[3]CREEC/CANECEV (CREES), MIVEGEC, Unité Mixte de Recherches, IRD 224–CNRS 5290–Université de Montpellier, Montpellier, France

[4]MTA-DE “Momentum” Ecology, Evolution & Developmental Biology Research Group, Dept. of Evolutionary Zoology, University of Debrecen, 4032 Debrecen, Egyetem tér 1, Hungary

[5]Faculty of Life Sciences, Core Facility for Cell Imaging and Ultrastructure Research,
University of Vienna, Djerassiplatz 1, 1030 Vienna, Austria

Corresponding author:

Astrid Collingro, Centre for Microbiology and Environmental Systems Science, University of Vienna, Djerassiplatz 1, 1030, Vienna, Austria; astrid.collingro@univie.ac.at

Content

1. Supplementary Figures

Supplementary Fig. 1. Relative abundance within chlamydiae in wild-derived *Hydra* polyps and water samples.
Supplementary Fig. 2. Average amino acid identity (AAI) values of CC-III proteomes and *Simkania negevensis*.
Supplementary Fig. 3. GC content variation in *E. hydrae* and *Parachlamydia acanthamoebae.*
Supplementary Fig. 4. Phylogenetic tree showing the relative evolutionary divergences (REDs) of each tree node.

2. Supplementary Text

Supplementary Text. Taxon description

3. Supplementary Tables

Supplementary Table 1. Presence of chlamydiae in *Hydra* individuals.
Supplementary Table 2. Presence of chlamydiae in the supernatant of *Hydra* cultures.
Supplementary Table 3. 16S rRNA gene counts in selected environments of the IMNGS database and hits against the *E. hydrae* sequence.
Supplementary Table 4. Genome sequences used in this study.
Supplementary Table 5. Annotation of the *E. hydrae* genome.
Supplementary Table 6. Relative abundance of chlamydiae in wild-derived *Hydra* polyps and water samples.
Supplementary Table 7. Prediction of biosynthetic gene clusters in *E. hydrae*.
Supplementary Table 8. Pseudogenes in the *E. hydrae* genome.
Supplementary Table 9. Transposases encoded in the *E. hydrae* genome.
Supplementary Table 10. *E. hydrae* genes in predicted genomic islands.
Supplementary Table 11. GC dynamics in the genomes of *E. hydrae* and *Parachlamydia acanthamoebae*.
Supplementary Table 12. OrthoFinder results.
Supplementary Table 13. Proteins with eukaryotic-like domains in *E. hydrae.*

4. Supplementary Data

Supplementary Data 1. Concatenated alignments of CheckM marker proteins for all genomes included in this study.
Supplementary Data 2. List of CheckM marker genes.
Supplementary Data 3. Chlamydial 16S rRNA gene sequences (OTUs) derived from screening of wild *Hydra* polyps and water samples.

5. Supplementary References

1. Supplementary Figures

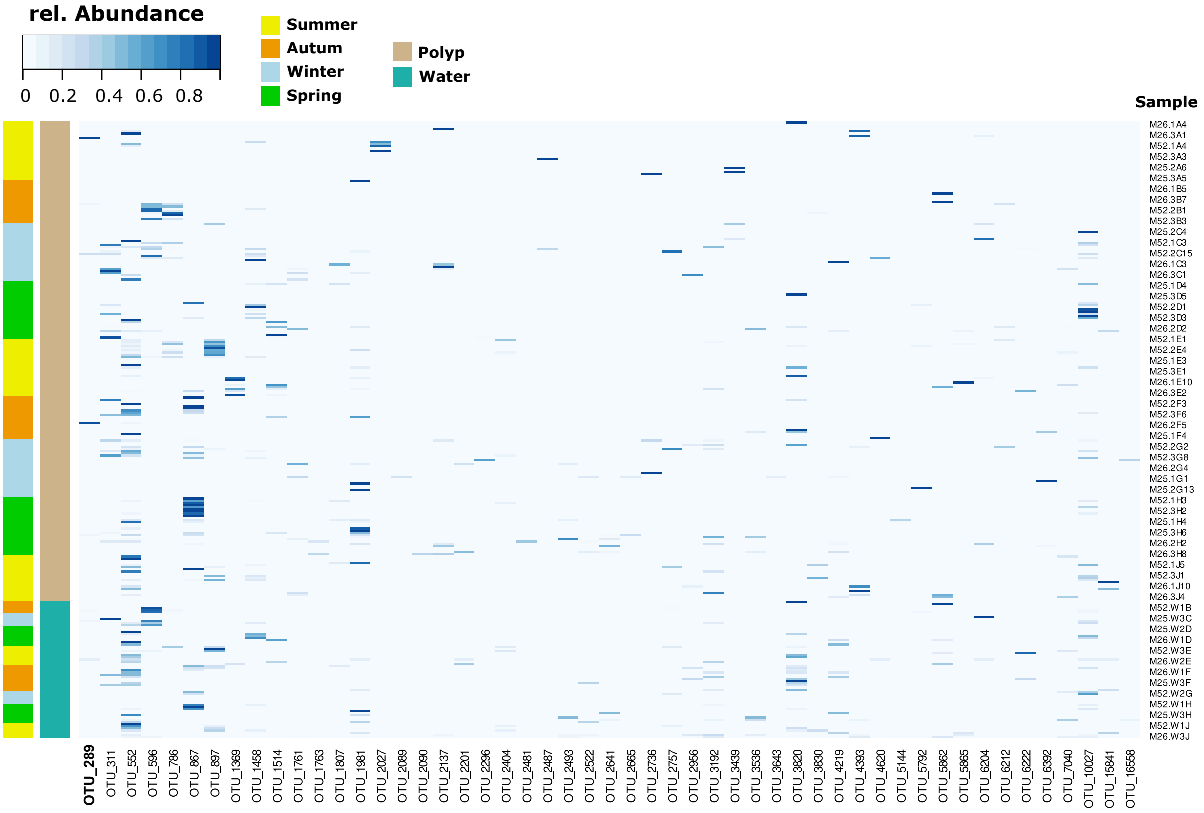

Supplementary Fig. 1. Relative abundance within chlamydiae in wild-derived *Hydra* polyps and water samples. Heatmap (using hierarchical clustering based on Euclidean distances) shows the abundance of each OTU relative to all chlamydial sequences, detected by sequencing of the V4 region of the 16S rRNA gene. In total 64 water and 287 polyp samples were collected from three distinct populations in Hungary between 2021 and 2023 in all four seasons. OTU 289 (bold) has >99% sequence identity to the *E. hydrae* 16S rRNA gene.

	3300012931–71	ZJ1.bin9	Jr–08jan18–52	SMAG_U4958	clean416	BP7.bin23	C15_metabat.bin.97	SZAS–17	clean353	clean375	1021942–14	*Endochlamydia hydrae*	SMAG_U15640	NC_groundwater_1613_48_32	REEB163	SMAG_U8262	VAR_78_metabat_106	CG10BFR821140104234	SMAG_U15670	SMAG_U15642	L024522	SSF52	REEB52	clean1594	3300009703–49	SIMNZ
3300012931–71	100	58	57	55	56	56	55	56	56	55	56	55	55	57	53	53	56	57	57	57	55	56	56	52	50	47
ZJ1.bin9	58	100	57	56	56	57	56	58	54	56	57	56	56	58	54	54	56	57	57	58	55	56	56	52	50	48
Jr–08jan18–52	57	57	100	61	61	59	58	60	57	58	59	59	58	60	55	56	60	60	61	61	57	59	59	53	51	49
SMAG_U4958	55	56	61	100	59	58	57	59	55	56	58	58	56	58	54	54	59	59	59	60	57	59	58	51	50	48
clean416	56	56	61	59	100	58	57	58	55	57	58	57	56	58	54	54	58	59	59	58	56	58	58	52	50	48
BP7.bin23	56	57	59	58	58	100	69	66	56	57	59	58	58	60	55	55	59	59	60	61	57	58	58	53	51	49
C15_metabat.bin.97	55	56	58	57	57	69	100	64	56	57	57	58	57	59	55	55	58	58	59	59	56	57	58	52	51	48
SZAS–17	56	58	60	59	58	66	64	100	56	58	60	59	58	61	55	56	60	60	62	62	59	60	60	53	51	48
clean353	56	54	57	55	55	56	56	56	100	62	59	57	57	58	54	55	57	58	58	58	56	57	57	51	50	47
clean375	55	56	58	56	57	57	57	58	62	100	61	59	58	60	56	56	59	58	60	59	57	59	59	52	51	48
1021942–14	56	57	59	58	58	59	57	60	59	61	100	61	59	61	57	57	61	61	62	62	58	60	60	53	51	49
Endochlamydia hydra	55	56	59	58	57	58	58	59	57	59	61	100	67	66	58	59	60	60	61	62	58	60	60	52	51	48
SMAG_U15640	55	56	58	56	56	58	57	58	57	58	59	67	100	64	57	58	59	59	60	61	57	58	59	51	50	48
NC_groundwater_1613_48_32	57	58	60	58	58	60	59	61	58	60	61	66	64	100	58	60	62	61	63	63	59	61	61	53	52	50
REEB163	53	54	55	54	54	55	55	55	54	56	57	58	57	58	100	64	56	55	56	56	54	55	55	51	49	48
SMAG_U8262	53	54	56	54	54	55	55	56	55	56	57	59	58	60	64	100	56	56	57	58	55	56	56	51	49	47
VAR_78_metabat_106	56	56	60	59	58	59	58	60	57	59	61	60	59	62	56	56	100	69	69	65	65	68	68	53	51	48
CG10BFR821140104234	57	57	60	59	59	59	58	60	58	58	61	60	59	61	55	56	69	100	69	65	64	67	66	52	52	48
SMAG_U15670	57	57	61	59	59	60	59	62	58	60	62	61	60	63	56	57	69	69	100	67	63	67	67	53	51	49
SMAG_U15642	57	58	61	60	58	61	59	62	58	59	62	62	61	63	56	58	65	65	67	100	61	64	63	53	52	49
L024522	55	55	57	57	56	57	56	59	56	57	58	58	57	59	54	55	65	64	63	61	100	63	63	52	51	48
SSF52	56	56	59	59	58	58	57	60	57	59	60	60	58	61	55	56	68	67	67	64	63	100	76	52	51	48
REEB52	56	56	59	58	58	58	58	60	57	59	60	60	59	61	55	56	68	66	67	63	63	76	100	52	51	49
clean1594	52	52	53	51	52	53	52	53	51	52	53	52	51	53	51	51	53	52	53	53	52	52	52	100	53	49
3300009703–49	50	50	51	50	50	51	51	51	50	51	51	51	50	52	49	49	51	52	51	52	51	51	51	53	100	48
SIMNZ	47	48	49	48	48	49	48	48	47	48	49	48	48	50	48	47	48	48	49	49	48	48	49	49	48	100

Supplementary Fig. 2. Average amino acid identity (AAI) values of CC-III proteomes and *Simkania negevensis*. AAI was calculated using the AAI calculator on http://enve-omics.ce.gatech.edu/aai/. Alignment options: Minimum length: 0aa, Minimum identity: 20%, Minimum score 0 bits, Minimum alignments 50. Each row and column represent a species, the value at the crosspoints show the respective AAI. Proteome sequences from *Endochlamydiaceae* and *S. negevensis* as closest related organism are shown (Supplementary Table 4).

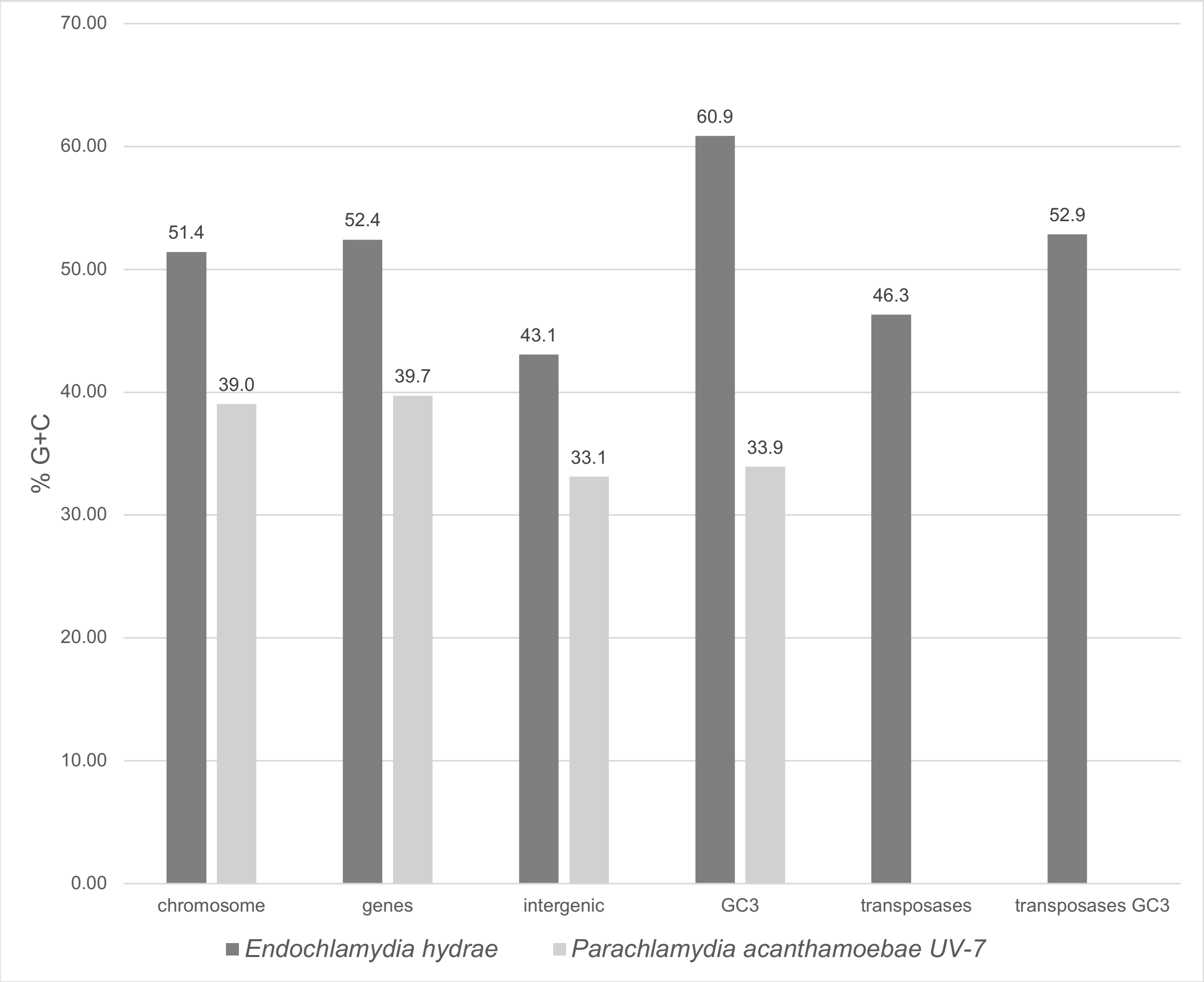

Supplementary Fig. 3. GC content variation in *E. hydrae* and *Parachlamydia acanthamoebae.* GC content in the complete chromosome compared to genes, intergenic regions, and at third codon positions of genes (GC3) are depicted for *E. hydrae* and a representative other chlamydial genome (*P. acanthamoebae* UV-7) (Supplementary Table 11). For *E. hydrae* GC content for transposase genes and their third codon positions are also included.

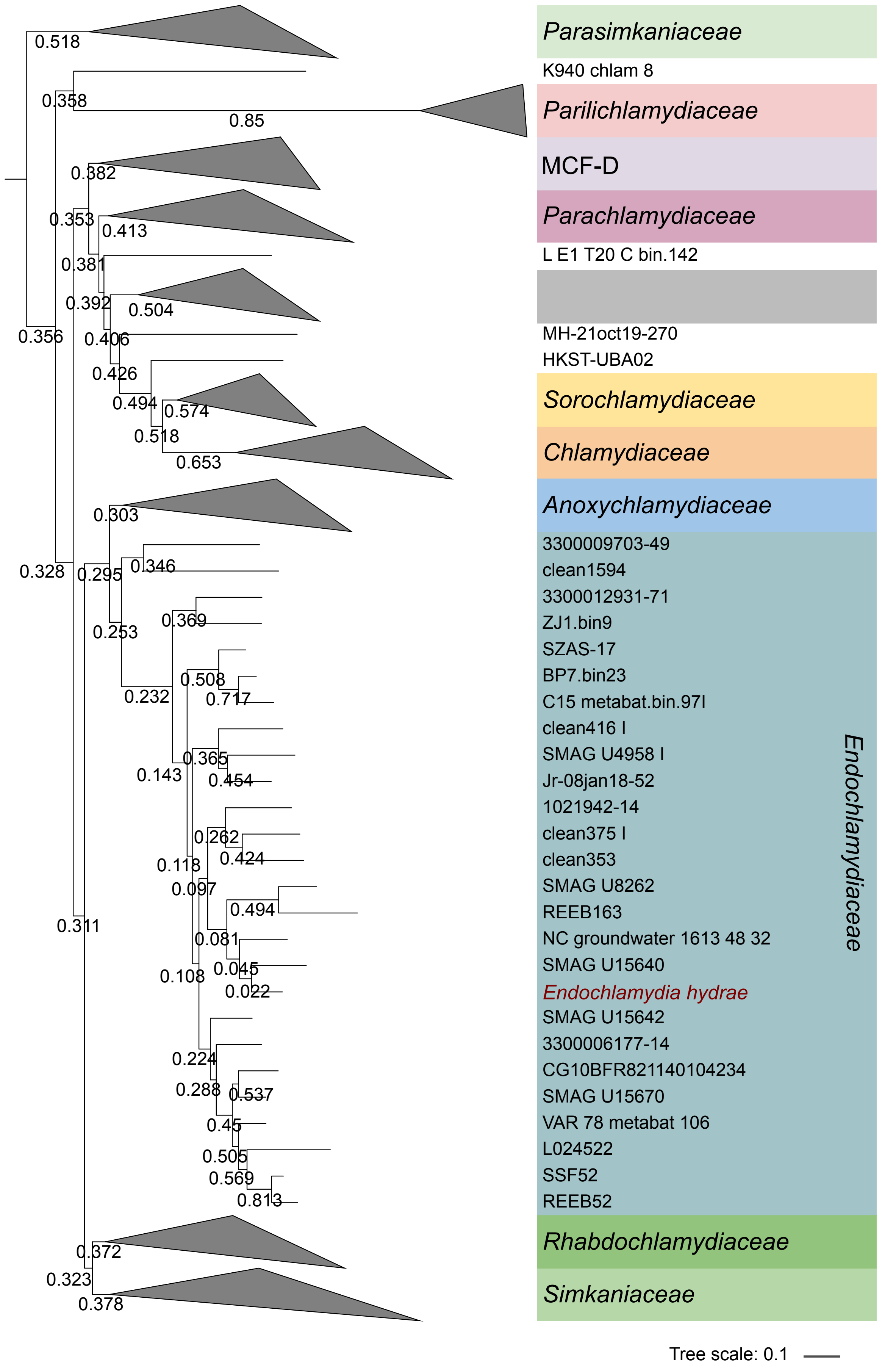

Supplementary Fig.4. Phylogenetic tree showing the relative evolutionary divergences (REDs) of each tree node. A maximum likelihood phylogenetic tree based on 43 conserved marker

proteins in 227 chlamydial genomes is shown. Support values based on 1 000 ultrafast bootstrap replicates and 1 000 replicates of the SH-like approximate likelihood ratio test are indicated on the branches. RED values are shown below each node. Scale bar represents 0.1 amino acid substitution per site.

2. Supplementary Text

Description of *Endochlamydiaceae* fam. nov.

En.do.chla.my.di.a.ce'ae. N. L. fem. n. *Endochlamydia* type genus of the family; *-aceae* ending to denote a family; N. L. fem. pl. n. *Endochlamydiaceae* the *Endochlamydia* family.

The family *Endochlamydiaceae* (formerly *Chlamydiae* Clade III) represents a distinct monophyletic lineage, sister to *Anoxychlamydiaceae*, supported by concatenated marker gene phylogenies in the present study (Fig. 5) and in prior work [1, 2]. *Endochlamydiaceae* members share conserved gene content and metabolism. Apart from *E. hydrae*, the family currently is based on metagenome-assembled genomes, their description and comparative analysis.

Description of *Endochlamydia*, gen. nov.

En.do.chla.my'di.a. Gr. adverb *endon* in/within; N. L. fem. n. *Chlamydia* taxonomic name of a bacterial genus; N. L. fem. n. *Endochlamydia* referring to the intracellular lifestyle of chlamydiae.

Type strain: *Endochlamydia hydrae*.

The genus *Endochlamydia* currently comprises the type strain and two related species described by metagenome-assembled genome sequences (MAGs), Chlamydiia bacterium NC_groundwater_1613_48_32 and Chlamydiales bacterium SMAG_U15640.
Based on phylogenetic analysis of marker genes (Fig. 5), average amino acid identity (Supplementary Fig. 2), and the 16S rRNA gene sequence they form a genus within the family *Endochlamydiaceae*. The currently closest cultured representative is *Simkania negevensis* Z (48% amino acid identity and 87.91% 16S rRNA gene identity).

Description of *Endochlamydia hydrae*, gen. nov., sp. nov.

N. L. fem. adj. *hydrae*; referring to the fresh-water cnidarian host *Hydra oligactis*.

Endochlamydia hydrae is the only cultivated representative within the family *Endochlamydiaceae*. Like other chlamydiae it has an obligate intracellular lifestyle with the typical biphasic developmental cycle inside large inclusions in endodermal cells of the *Hydra* host. It was discovered in *H. oligactis* polyps isolated from a freshwater pond (Montaud, France). The symbionts are non-motile and are not cultivable outside eukaryotic host cells but can also successfully replicate in *Spodoptera frugiperda* (Sf9) insect cell lines. The morphology of *Endochlamydia hydrae* resembles that of most chlamydiae with two distinct morphological forms. Replicating reticulate bodies are round and about 500-800 nm in diameter and infectious non-replicating elementary bodies are elongated and about 300-500 nm in length. The complete genome sequence is available under the accession number PRJEB86254.

5. Supplementary References

1. Dharamshi JE, Tamarit D, Eme L, et al. Marine sediments illuminate chlamydiae diversity and evolution. *Curr Biol* 2020;**30**:1032-1048.e7. https://doi.org/10.1016/j.cub.2020.02.016

2. Dharamshi JE, Köstlbacher S, Schön ME, et al. Gene gain facilitated endosymbiotic evolution of Chlamydiae. *Nat Microbiol* 2023;**8**:40–54. https://doi.org/10.1038/s41564-022-01
